# A Case of Pleuritis Associated With Antineutrophil Cytoplasmic Antibody (ANCA)-Associated Vasculitis Diagnosed Through Medical Thoracoscopy

**DOI:** 10.7759/cureus.69827

**Published:** 2024-09-21

**Authors:** Tomohiro Sakamoto, Kohei Yamane, Yasuhiko Teruya, Tomoya Harada, Akira Yamasaki

**Affiliations:** 1 Division of Respiratory Medicine and Rheumatology, Department of Multidisciplinary Internal Medicine, Faculty of Medicine, Tottori University, Yonago, JPN

**Keywords:** anca, anca-associated vasculitis (aav), pleural effusion, pleuritis, thoracoscopy under local anesthesia

## Abstract

Antineutrophil cytoplasmic antibody (ANCA)-associated vasculitis (AAV) includes eosinophilic granulomatosis with polyangiitis, granulomatosis with polyangiitis, and microscopic polyangiitis. Pulmonary involvements such as interstitial pneumonia and alveolar hemorrhage are common in AAV, but pleuritis is rare. Here, we report a case of pleuritis associated with AAV. A 68-year-old woman was referred to our hospital because of bilateral wrist and knee pain, Raynaud’s phenomenon, and a sclerotic change in her extreme fingers with elevation of proteinase 3 ANCA (PR3-ANCA) and myeloperoxidase-ANCA (MPO-ANCA). From a skin biopsy of her forearms and fingers, we diagnosed that the patient had limited systemic sclerosis. After her first visit at 15 months, she complained of pain in the side of her chest. Her chest X-ray and computed tomography showed left pleural effusion, and local anesthetic thoracoscopy was performed. Histological examination of the pleural revealed granuloma and vasculitis. Based on her symptoms and histological findings, we diagnosed this case as ANCA-associated vasculitis (AAV) and treated it with steroids and intravenous cyclophosphamide successfully. Pleuritis is a rare pulmonary lesion of AAV, and thoracoscopy under local anesthesia is useful for the histological examination of vasculitis.

## Introduction

Differential diagnosis of pleural effusion is usually determined through cytology and bacterial culture of the pleural effusion, cell differentiation, and measurement of lactate dehydrogenase (LDH) levels, total protein, adenosine deaminase (ADA), and hyaluronic acid in the pleural effusion. However, it is not easy to diagnose accurately by thoracentesis [1]. Thoracoscopy has been used for the diagnosis and treatment of several diseases, including malignant pleural effusion, malignant pleural mesothelioma, tuberculous pleural effusion, empyema, complicated parapneumonic effusion, and pneumothorax [2]. The usefulness of thoracoscopy under local anesthesia was first reported in 1979 [3]. It has since become a safely and widely performed procedure and has been a valuable means to diagnose pleural disease with effusion globally, including in Japan, around the 1990s.

Pulmonary and pleural involvement of antineutrophil cytoplasmic antibody (ANCA)-associated vasculitis (AAV) is primarily alveolar hemorrhage and interstitial pneumonia. Although pleuritis is also observed in AAV, this fact is slightly underestimated in clinical practice [4]. As a result, there are quite few reports examining the pathology of pleuritis in AAV. It has reported that when unrecognized as a manifestation of AAV, pleuritis and pericarditis frequently were treated in terms of symptoms and led to recurrences or further organ involvement of AAV [4]. This morbidity potentially may be avoided if AAV is considered in the differential diagnosis of pleuritis and pericarditis and treated with immunosuppression. Additionally, the accurate diagnosis of AAV with untypical symptoms is sometimes challenging when the manifestation of AAV is nonspecific with no prominent histological findings. This article reports a case of pleuritis related to AAV diagnosed by thoracoscopy under local anesthesia.

## Case presentation

A 68-year-old woman was referred to our hospital because of bilateral wrist and knee pain, Raynaud’s phenomenon, and a sclerotic change in her distal fingers. She had been treated with propylthiouracil (PTU) for 10 years because of hyperthyroidism. Physical examination revealed mild sclerotic changes in all fingers from the distal interphalangeal joint.

The chest examination revealed fine crackles in her bilateral back lung fields. However, chest X-ray and computed tomography images revealed no fibrotic change. The antinuclear antibody test was positive (1:320, centromere pattern), the proteinase 3 ANCA (PR3-ANCA) level was 37.2 U/mL, and the myeloperoxidase-ANCA (MPO-ANCA) level was 15.2 U/mL. Skin biopsy of her forearms and fingers revealed thickening and sclerosis of the dermic layer without vasculitis. The initial chest computed tomography did not observe fibrosis, consolidation, or pleural effusion. Therefore, we diagnosed the patient as having limited systemic sclerosis.

**Figure 1:**
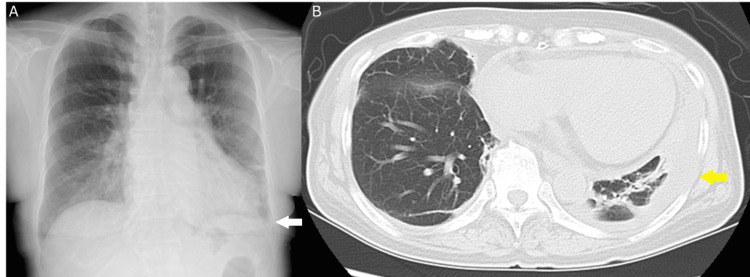
Chest X-ray and non-contrast-enhanced computed tomography (CT) of the lung (A) The chest X-ray image shows pleural effusion in the left lung. (B) The chest CT scan shows pleural effusion in the left lung.

Approximately 15 months after the initial visit, she complained of arthralgia of the foot, knee, and shoulder and left chest pain. She visited our hospital’s emergency department. Her chest X-ray and computed tomography images showed left pleural effusion (Figure 1, Panels A and B). The administration of oral antibiotics was ineffective.

She was admitted to our hospital for further examination and treatment. The puncture of pleural effusion showed an exudate with a glucose level of 102 mg/dl, ADA of 31.6 U/L, and an increased number of inflammatory cells (13,430/mL), predominantly neutrophilia (87%), but no bacteria or fungi were detected. The serum PR3-ANCA and MPO-ANCA levels were 32.4 U/mL and 19.8 U/mL, respectively.

**Figure 2:**
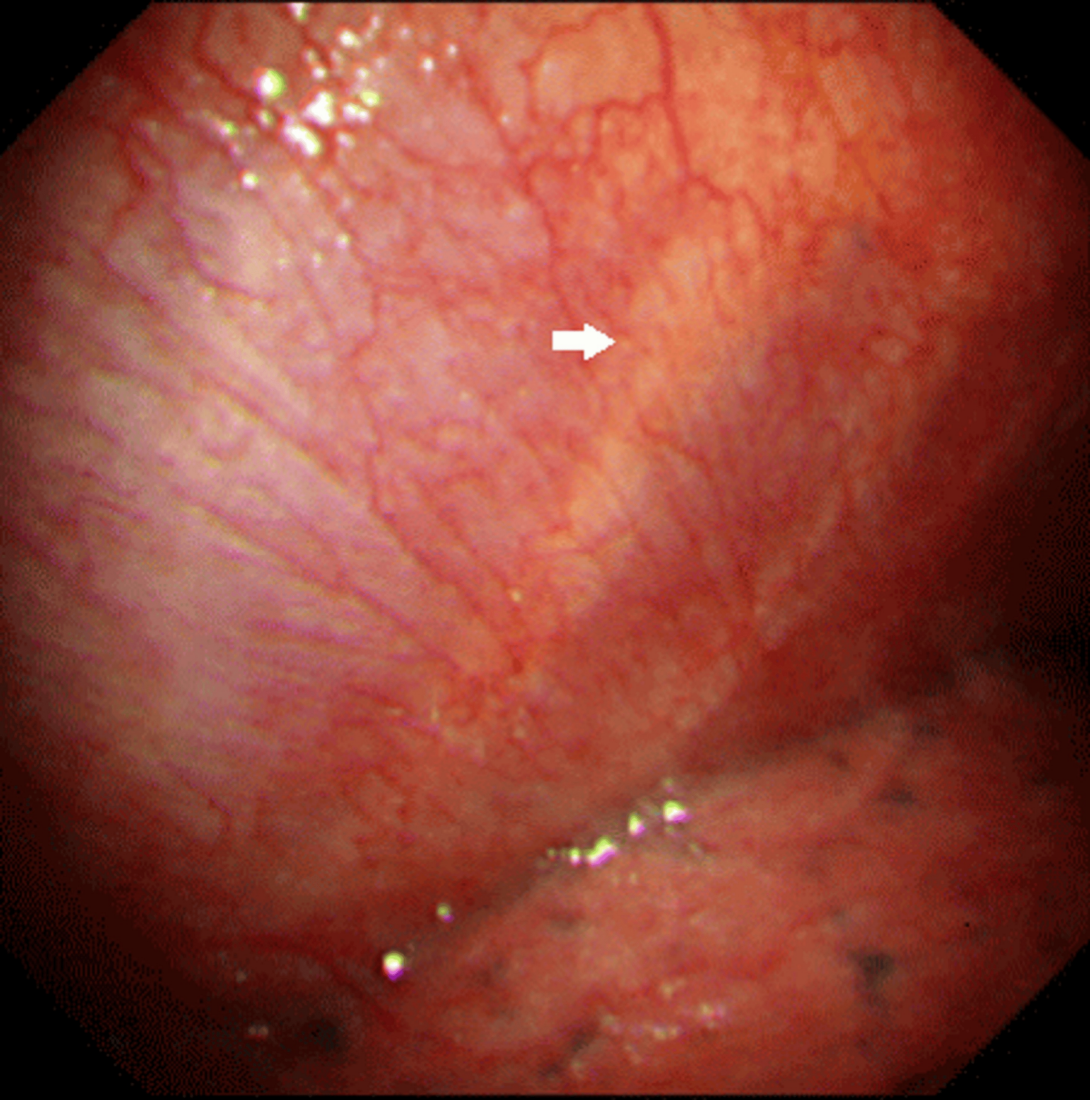
The thoracoscopy finding Thickening of the visceral and pulmonary pleura due to fibrin was observed. The costal bones were seen through the parietal pleura.

**Figure 3:**
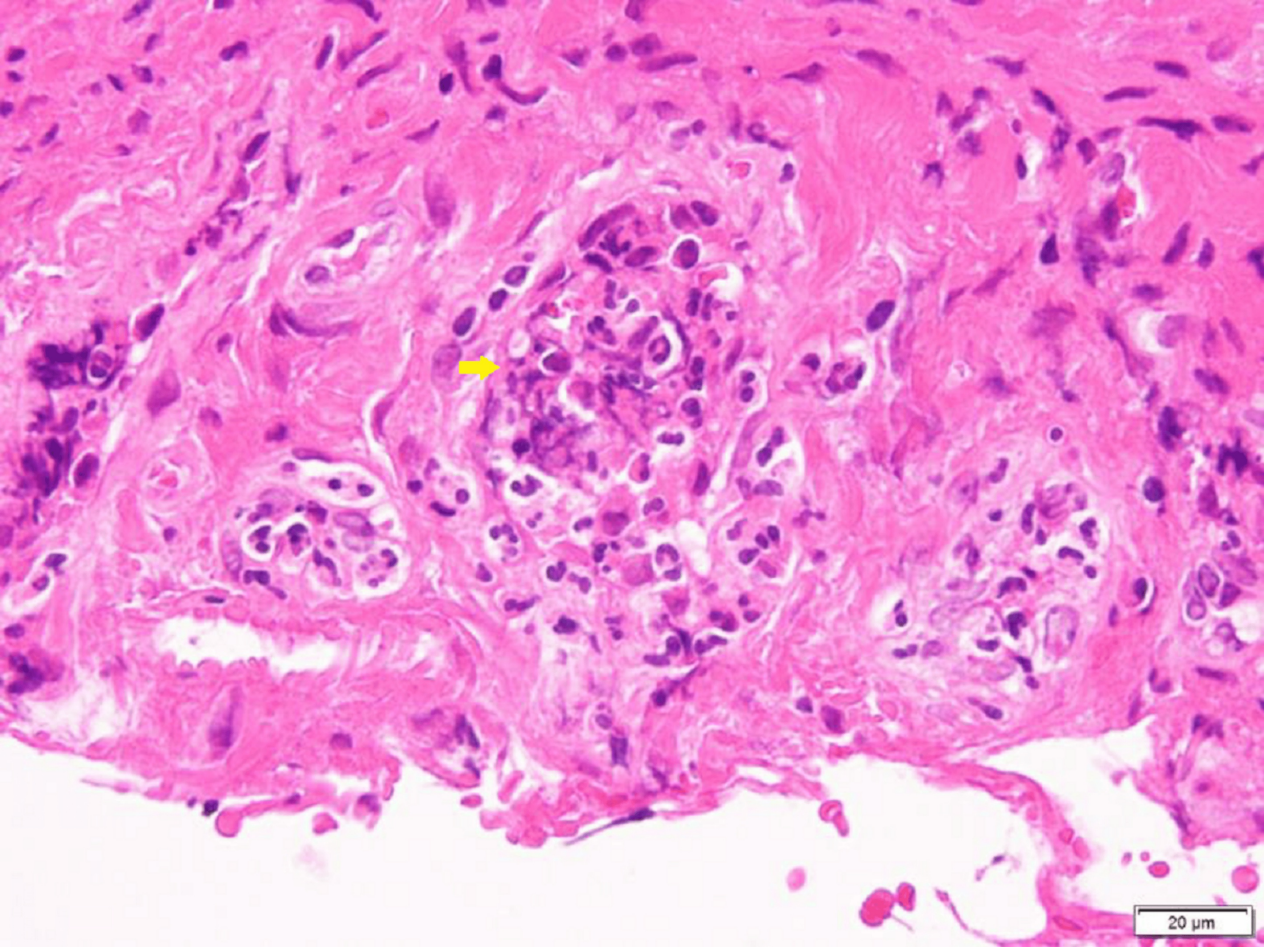
Hematoxylin and eosin staining of the visceral pleura The destruction of the vessel wall with infiltration of inflammatory cells, predominantly neutrophils, lymphocyte macrophages, and neutrophils, with foaming granuloma were observed.

Pleuritis associated with connective tissue disease was suspected. Therefore, thoracoscopy was performed under local anesthesia. The thoracoscopy findings were thickening of the visceral and pulmonary pleura with fibrin (Figure 2). Hematoxylin and eosin staining of the visceral pleura showed the destruction of the vessel wall with infiltration of inflammatory cells, predominantly neutrophils, lymphocyte macrophages, and neutrophils, with foaming granuloma (Figure 3).

The renal biopsy results and magnetic resonance imaging of the brain and paranasal sinus revealed no AAV findings. During the admission period, right pleural effusion and pericardial effusion appeared. A diagnosis of AAV was determined based on her symptoms, clinical data, and histological findings from pleural biopsy. PTU was stopped because drug-induced AAV was suspected. She was administered systemic therapy with steroids (i.e., methylprednisolone, 500 mg/day for three days, following oral prednisolone) and cyclophosphamide pulse therapy. Her symptoms and bilateral pleural effusion disappeared; therefore, she was discharged from the hospital. She continued treatment with a tapering dose of oral prednisolone. The PR3-ANCA level was decreased to 4.0 U/mL, whereas the MPO-ANCA level was unchanged (16.2 U/mL) after one year of treatment with prednisolone.

## Discussion

Differential diagnosis of pleural effusion is sometimes tricky based on examination of pleural effusion from a puncture or blind pleural biopsy [5]. Flores-Franco et al. [6] proposed a decision tree of exudate acidosis with a low glucose level to diagnose systemic vasculitis. For our patient, the finding of pleural effusion with average glucose level made determining a diagnosis difficult. However, we successfully diagnosed this case as pleuritis associated with AAV by thoracoscopy under local anesthesia.

Thoracoscopy is safe and a gold standard for diagnosing pleural disease and has high sensitivity and specificity for diagnosing malignant disease [7,8]. Since the 1990s, flexible thoracoscopy has been developed instead of rigid thoracoscopy; surgeons and physicians can perform this procedure with local anesthesia. The usefulness of thoracoscopy under local anesthesia has been reported in other pleural diseases [9,10]. Furthermore, Gokce et al. reported that awake video-associated thoracoscopy surgery (VATS) with local anesthesia has a similar diagnostic efficacy and safety profile to VATS with general anesthesia [11].

ANCA-associated vasculitis is classified as eosinophilic granulomatosis with polyangiitis, granulomatosis with polyangiitis, microscopic polyangiitis, and unclassified AAV. We diagnosed the patient with AAV based on the findings of vasculitis of the pleura and no granuloma in the paranasal space, no glomerulonephritis in the kidney biopsy, and no allergic disease with eosinophilia. Several cases of PTU-induced AAV have been reported since the 1990s, and approximately 15%-64% of patients taking PTU become ANCA-positive [12].

Since the patient has been taking PTU for several years, we diagnosed this case as PTU-induced AAV. According to the Watt algorithm, PTU-induced AAV should be excluded [13]. PTU-induced AAV is mild and has a good prognosis compared to primary AAV; however, some patients exhibit pulmonary hemorrhaging or rapidly progressive glomerulonephritis [12]. Rare manifestations of PTU-induced AAV have been reported, such as nasal septal perforation [14] or mimic Kawasaki’s disease [15]. Limited systemic sclerosis may be associated with the development of AAV because approximately 10% of patients with systemic sclerosis are ANCA-positive [16]. Thoracoscopy helped determine a diagnosis of vasculitis. However, few patients present with AAV [16]. Rare manifestations of PTU-induced AAV have been reported, such as nasal septal perforation [14] or mimic Kawasaki’s disease [15].

## Conclusions

This case report describes a patient with pleuritis caused by AAV. Local anesthetic thoracoscopy was useful for determining a diagnosis. Pleuritis is a relatively rare manifestation of AAV. However, thoracoscopy under local anesthesia may be considered in suspected cases of AAV with pleural involvement or in patients taking drugs that induce AAV.
